# Simultaneous Processing of Information on Multiple Errors in Visuomotor Learning

**DOI:** 10.1371/journal.pone.0072741

**Published:** 2013-08-29

**Authors:** Shoko Kasuga, Masaya Hirashima, Daichi Nozaki

**Affiliations:** 1 Faculty of Science and Technology, Keio University, Yokohama, Japan; 2 Graduate School of Education, The University of Tokyo, Tokyo, Japan; 3 Research Fellow of the Japan Society for the Promotion of Science, Tokyo, Japan; VU University Amsterdam, Netherlands

## Abstract

The proper association between planned and executed movements is crucial for motor learning because the discrepancies between them drive such learning. Our study explored how this association was determined when a single action caused the movements of multiple visual objects. Participants reached toward a target by moving a cursor, which represented the right hand’s position. Once every five to six normal trials, we interleaved either of two kinds of visual perturbation trials: rotation of the cursor by a certain amount (±15°, ±30°, and ±45°) around the starting position (single-cursor condition) or rotation of two cursors by different angles (+15° and −45°, 0° and 30°, etc.) that were presented simultaneously (double-cursor condition). We evaluated the aftereffects of each condition in the subsequent trial. The error sensitivity (ratio of the aftereffect to the imposed visual rotation) in the single-cursor trials decayed with the amount of rotation, indicating that the motor learning system relied to a greater extent on smaller errors. In the double-cursor trials, we obtained a coefficient that represented the degree to which each of the visual rotations contributed to the aftereffects based on the assumption that the observed aftereffects were a result of the weighted summation of the influences of the imposed visual rotations. The decaying pattern according to the amount of rotation was maintained in the coefficient of each imposed visual rotation in the double-cursor trials, but the value was reduced to approximately 40% of the corresponding error sensitivity in the single-cursor trials. We also found a further reduction of the coefficients when three distinct cursors were presented (e.g., −15°, 15°, and 30°). These results indicated that the motor learning system utilized multiple sources of visual error information simultaneously to correct subsequent movement and that a certain averaging mechanism might be at work in the utilization process.

## Introduction

In order to control limb movements in various environments, the brain learns to construct a neural feedforward controller that is known as the internal model [1]–[4]. Movement errors are the driving force of modifications of the motor commands in this learning process; therefore, information about such errors should be appropriately assigned to the movement controller in order to correct the motor command. The problem of how the brain associates motor actions with their consequences is called the credit assignment problem, which has been thoroughly investigated [5]–[7]. Conventional studies have dealt with the case in which an action (i.e., reaching with one arm) causes its consequence (i.e., a single-cursor error) in a one-to-one manner, but this is not always true. For example, during a visuomotor learning task in which participants move a cursor bimanually (e.g., when the cursor is positioned between the hands), they perform two distinct actions with each arm but receive only one movement error. In this situation, the movements of both arms adapt to the visuomotor rotation that is imposed on the cursor [8], [9].

The adaptation of movement by both arms to such visuomotor rotation during bimanual movements is also observable when participants have explicit knowledge that the cursor’s movement is associated with only one hand but they do not notice the rotation because it occurs gradually [8]. This implies that the error information is implicitly processed in the motor learning system [10] and used for automatic correction of the movement controllers of both arms.

In order to explore this intriguing ability of the motor learning system further, we sought to find out how the motor learning process occurs in a visuomotor task in which a reaching movement created multiple visual outcomes. For example, when two cursors that are being moved simultaneously with one hand show different directional movement errors due to different degrees of visual rotation, is it that only one cursor utilizes the movement controller while the other is ignored or is it that the perceived error is determined by the averaged direction of the two cursors? Alternatively, is the information from all observed cursors used concurrently in different manners to modify the subsequent motor command? In a previous study [8], we have observed that when participants bimanually move two cursors, each of which represents the position of a hand, the movement of each hand is corrected according to not only the error of the cursor that is moved by the hand, but also the error of the cursor that is moved by the other hand (i.e., movement in one hand is simultaneously affected by two cursors). Considering this observation and the implicit nature of visuomotor learning, we hypothesized that multiple visual errors are utilized simultaneously by the movement controller. In this study, we examined this hypothesis and how the information of each error was utilized in the visuomotor learning process.

## Materials and Methods

### Ethics Statement

This study was conducted according to the Declaration of Helsinki. The experimental procedures were approved by the ethics committee of the Graduate School of Education at the University of Tokyo. Written informed consent was obtained from all participants prior to the experiments.

### Participants

Forty-four neurologically normal individuals (11 women and 33 men, aged 19–39 years) participated in the experiments. All participants, except one, were right-handed (mean ± standard error [SE] of laterality quotient [LQ] = 84.6±3.2), as assessed by the Edinburgh Handedness Inventory [11]. They were randomly assigned to one of three experimental groups (18, 13, and 13 participants in experiments 1, 2, and 3, respectively), and each participant was included in only one experiment. All participants were naïve to the purpose of the experiments.

### Apparatus

**Figure 1:**
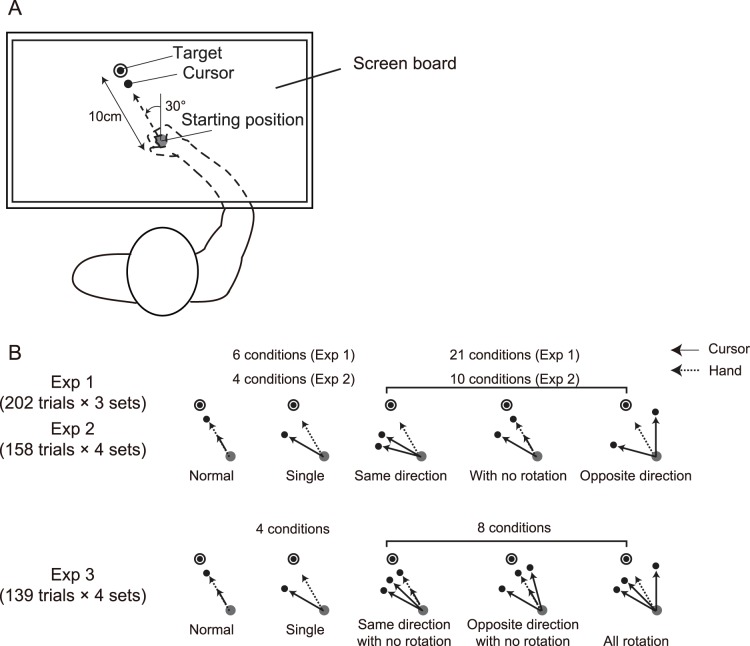
Experimental setup and protocols of visual perturbation by multiple cursors. A: Visual information was displayed on a horizontal white screen board above the hand. Double circles indicate targets, gray circles indicate starting positions, and black circles indicate cursors on the screen. B: In normal trials, a cursor followed the actual movement of the handle (“Normal”), whereas in the single-cursor trials, the cursor was rotated around the starting position (“Single”). In the double-cursor trials of experiments 1 and 2, two cursors were rotated in the same direction (“Same direction”) to different degrees or in the opposite direction (“Opposite direction”) or one cursor was not rotated (“With no rotation”). In the triple-cursor trials (experiment 3), two of three cursors were rotated in the same direction (“Same direction with no rotation”) or the opposite direction (“Opposite direction with no rotation”) or all cursors were rotated in either direction (“All rotation”).

The experiments were performed in a darkened room. The participants sat on a straight-backed chair while grasping the handle of a robotic manipulandum (Phantom Premium 1.5 HF, Geomagic, Morrisville, NC, USA) with their right hand. A virtual spring that was simulated by the Phantom device (1.0 N/mm) generated a virtual horizontal plane on which the handle could be moved. A projector was used to display the position of the handle (indicated by a 6-mm-diameter white circle cursor) on a horizontal screen (45 cm × 60 cm) that was placed approximately 13 cm above the virtual plane and about 15 cm below the shoulder level. Thus, the screen board prevented the participants from directly seeing their arm and the handle. The participants controlled the cursor by performing reaching movements from a starting position (10 mm diameter) and moving toward a target (10 mm diameter), which was also displayed on the screen. The starting position was located approximately 25 cm in front of the body in the midsagittal plane, and the target was 10 cm away from it and rotated counterclockwise by 30° (Fig. 1A). The starting point and cursor were always visible. The position of the handle was converted by an analog-to-digital converter (sampling frequency, 500 Hz) and stored for offline analysis.

### Procedure

The participants were instructed to move the cursor from the starting position to the target by performing straight, fast, and uncorrected reaching movements. Before each trial, they had to place the cursor at the starting point, and then the gray target appeared 2 s later. After an additional randomly selected holding time (1–2 s), the target color changed (the ‘go’ cue). In all tasks, they were asked to perform a movement within 2 s after the target’s appearance. They were also asked to maintain the peak velocities as constantly as possible across the trials. In order to facilitate this, a warning message was presented on the screen if the speed of either handle was above (Fast) or below (Slow) the target speed range of 470±45 mm/s. The trial was ended when 2 s passed after the ‘go’ cue, and then the target disappeared and the robotic manipulandum automatically moved the handle to the starting position for the next trial. There were 35–45 practice trials with normal visuomotor conditions and varying patterns of rotation before each experiment.

### Experiment 1: Double-cursor Condition for Large Rotations

This experiment was designed to examine how the brain processes two simultaneously observed visual movement errors. The experiment consisted of three sets of trials, and each set consisted of 202 trials (606 trials in total). Once every five to six trials, the following visual perturbation trials were interleaved (Fig. 1B): the cursor was rotated around the starting position (±15°, ±30°, and ±45°; six patterns) in the single-cursor trials, and two cursors were displayed in the double-cursor trials, and both were rotated by different angles (0°, ±15°, ±30°, and ±45°; 21 patterns). Note that a rotation of 0° meant that the cursor followed the actual movement of the handle (i.e., no rotation). The participants performed 27 patterns of perturbation trials (six patterns for the single-cursor trials and 21 patterns for the double-cursor trials), four times each. In summary, 108 out of 606 trials were perturbation trials (24 trials for the single-cursor trials and 84 trials for the double-cursor trials). We defined the first four trials of each set (12 trials in total), which occurred before the first introduction of visual perturbation and thus had no aftereffects from the perturbation, as baseline trials. The cursors were always visible to the participants throughout the experiments.

### Experiment 2: Double-cursor Condition for Small Rotations

According to the findings of a previous study [12], the degree of adaptation depends on the degree of imposed visual rotation. Consequently, depending on the range of imposed visual rotations, the experiment described above may not always yield observable results. Therefore, we performed another experiment (experiment 2), which was designed to induce results when the imposed visual rotations were smaller.

As in experiment 1, we interleaved the following visual perturbation trials once every five to six trials (Fig. 1B): the cursor was rotated around the starting position (±5° and ±10°; four patterns) in the single-cursor trials, and two cursors that were rotated by different angles were presented (0°, ±5°, and ±10°; 10 patterns) in the double-cursor trials. Experiment 2 consisted of four sets of trials, and each set consisted of 158 trials (632 trials in total). The participants performed 14 patterns of perturbation trials (four patterns for the single-cursor trials and 10 patterns for the double-cursor trials) eight times each, and the number of unperturbed interval trials was randomized. In summary, 112 out of 632 trials were perturbation trials (32 trials for the single-cursor trials and 80 trials for the double-cursor trials).

### Experiment 3: Triple-cursor Condition

Experiment 3 was designed to examine how the movement control process utilized errors when the number of cursors was increased. As in experiment 1, the following visual perturbation trials were interleaved once every five to six trials (Fig. 1B): the cursor was rotated around the starting position (±15° and ±30°; four patterns) in the single-cursor trials, and three cursors that were rotated by different angles were presented (0°, ±15°, and ±30°; eight patterns in total) in the triple-cursor trials. We excluded combinations of −15°, 0°, and 15° and −30°, 0°, and 30° because the results of experiment 1 showed that we could predict a small amount of aftereffects, if any, for these combinations because the aftereffects from the two degrees of rotation that were the same but in opposite directions would be counterbalanced. The experiment consisted of four sets of trials, and each set consisted of 139 trials (456 trials in total). The participants performed 12 patterns of perturbation trials (four patterns for the single-cursor trials and eight patterns for the double-cursor trials) eight times each, and the number of unperturbed interval trials was randomized. In summary, 96 out of 456 trials were perturbation trials (32 trials for the single-cursor trials and 64 trials for the double-cursor trials).

### Quantification of Aftereffects

The position data of the handle was low-pass filtered with a zero-lag fourth-order Butterworth filter (5-Hz cutoff). The velocity of the handle was then calculated by differentiating the position data with a three-point central difference equation. We recorded the position at which the outward velocity peaked. The movement direction was defined as the angle between a line connecting the starting position and the target and a line connecting the starting position and the position where the peak velocity was observed (167.5±4.4 ms after movement onset in experiment 1, for example). The clockwise (CW) and counter-clockwise (CCW) directions were defined as the negative and positive values of the direction, respectively. The aftereffect of each visual perturbation was defined as the movement direction of the trial just after the visual perturbation trial minus the averaged value of the movement direction of the baseline trials.

### Data Analysis

Before the statistical analyses, we precluded trials in which the aftereffects exceeded the mean ±2 SD of each cursor combination pattern. In order to quantitatively investigate the sensitivity to which the error was corrected in the subsequent trial, we calculated the ratio (*K_s_*) of the aftereffect (*y*) to each observed error (*r*) in the single-cursor trials, as follows:
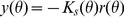
(1)where*θ* is the imposed visual rotation (±15°, ±30°, and ±45° in experiment 1; ±5° and ±10° in experiment 2; and ±15° and ±30° in experiment 3). The observed error (*r*) was defined as the angular difference between the cursor direction and the target direction at the peak velocity point in each trial. Note that *r*(*θ*) was almost identical to *θ* in our experiment, and the calculation of *K_s_* with *θ* instead of *r*(*θ*) did not substantially change the results. It should also be noted that in eq. (1), *K_s_*(*θ*) is represented as a function of *θ* because it should depend on the amount of visual rotation [12]. *K_s_*(*θ*) was calculated for each participant with the averaged aftereffect from every pattern of imposed visual rotation in the single-cursor trials (six patterns in experiment 1, four patterns in experiment 2, and four patterns in experiment 3).

Next, in order to investigate how the information from the cursors was integrated, we assumed that *y* was represented as the linear function of *r* where the imposed visual rotations are indicated by *θ*_1_ and *θ*_2_ :
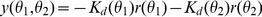
(2)where *θ*_1_ and *θ*_2_ = 0°, ±15°, ±30°, and ±45° in experiment 1 and 0°, ±5°, and ±10° in experiment 2. Note that *θ*_1_ and *θ*_2_ do not indicate a rotation of a particular cursor and thus can be an arbitrary rotation. Furthermore,
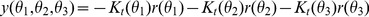
(3)where *θ*_1_, *θ*_2_, and *θ*_3_ = 0°, ±15°, and ±30°, respectively, in experiment 3.

We estimated a set of parameters, *K_d_* and *K_t_*, with the least-squares method. The estimation was performed for the data from individual participants. The same analysis was also performed for the averaged data from all participants, but the results were not substantially different. Specifically, for each participant, we prepared a dataset of the averaged aftereffect from every pattern (i.e., cursor combination) in the double-cursor trials (21 data points in experiment 1 and 10 data points in experiment 2) or the triple-cursor trials (eight data points). With the dataset, we estimated the parameters, *K_d_* or *K_t_*, for the imposed visual rotations (six parameters in experiment 1, four parameters in experiment 2, and four parameters in experiment 3).

### Statistics

The data values are expressed as means ± SE. One-sample *t*-tests were used to examine whether the aftereffects were greater or smaller than 0. Paired two-samples *t*-tests were performed in order to detect significant differences between the aftereffects of different cursor combinations. A linear regression between the estimated parameters (*K_d_* and *K_t_*) and the error sensitivities of the single-cursor trials (*K_s_*) was used to examine the relationships of the variables and the calculated confidence intervals (CI) of the coefficients. Two-way repeated measures or oneone-way analysis of variance (ANOVA) was performed for the values from experiments 1, 2, or 3 in order to investigate the differences between the estimated parameters as well. If a significant difference was detected by ANOVA, a post hoc Bonferroni test was used for multiple comparisons. Two-way repeated measures ANOVA was used to analyze an asymmetry in *K_s_* that depended on the direction of rotation. The significance threshold was set at *P<*0.05.

## Results

### Experiments 1 and 2

#### Aftereffects and their ratios to the rotations in the single-cursor condition

**Figure 2:**
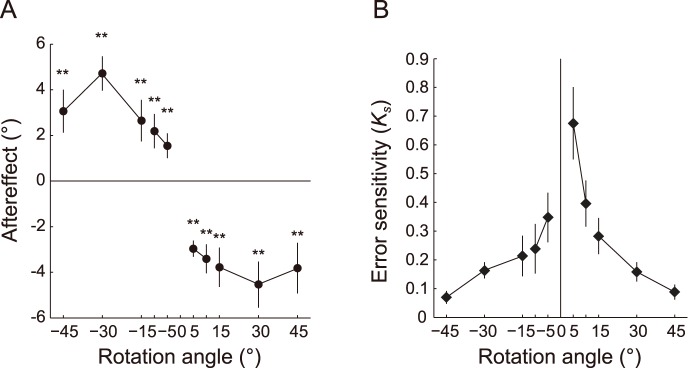
Results of the single-cursor trials in experiments 1 and 2. Both experiments are shown in the same panel, but the lines are disconnected between 10° and 15° because the data originated from different experiments. The data from −45° to −15° and 15° to 45° were adopted from experiment 1 and those from −10° to 10° were adopted from experiment 2. A: Aftereffects of the single-cursor trials for each rotation. The asterisks indicate significant directional shifts from baseline (**P<0.01). B: The error sensitivity to the imposed rotations (*K_s_*) decayed as the magnitude of rotation increased. The error bars indicate ±1 SE.

First, we examined how the movement control process adapted, in the subsequent trial, to a sudden error. In the single-cursor trial, significant aftereffects to all visual rotations were observed in both experiments 1 and 2 (Table 1 and Table 2; Fig. 2A). Overall, the aftereffects were not proportional to the imposed visual rotations (i.e., the magnitude of the error in the previous trial); as the imposed rotations became larger, the aftereffects tended to be saturated. However, when the imposed rotations were relatively small, the aftereffects appeared to increase with the imposed rotations.

**Table 1:** Aftereffects of all the single- and double-cursor trials in experiment 1.

Rotation (°)	−45	−30	−15	0	15	30	45
−45	3.07** (0.62)	3.16** (0.66)	2.86** (0.58)	1.47* (0.71)	0.58 (0.57)	1.00 (0.53)	0.93 (0.55)
−30	3.16** (0.66)	4.69** (0.60)	2.49** (0.66)	2.40** (0.61)	0.96 (0.66)	1.16 (0.74)	0.91 (0.64)
−15	2.86** (0.58)	2.49** (0.66)	2.64** (0.62)	1.53** (0.55)	1.31* (0.62)	−0.26 (0.51)	−0.48 (0.57)
0	1.47* (0.71)	2.40** (0.61)	1.53** (0.55)	0	0.20 (0.55)	−0.62 (0.59)	−1.41* (0.56)
15	0.58 (0.57)	0.96 (0.66)	1.31* (0.62)	0.20 (0.55)	−3.80** (0.63)	−3.85** (0.56)	−4.24** (0.56)
30	1.00 (0.53)	1.16 (0.74)	−0.26 (0.51)	−0.62 (0.59)	−3.85** (0.56)	−4.59** (0.68)	−4.92** (0.55)
45	0.93 (0.55)	0.91 (0.64)	−0.48 (0.57)	−1.41* (0.56)	−4.24** (0.56)	−4.92** (0.55)	−3.85** (0.67)

The cell at the junction of the row labeled −15 and the column labeled 30 shows the aftereffect of a double-cursor trial when two cursors were rotated by −15° and 30°. The cell at the junction of the row labeled −45 and the column labeled −45 shows the aftereffect of a single-cursor trial when the cursor was rotated by −45°. The upper value of each cell indicates the mean and the value in parentheses indicates 1 SE. The asterisks indicate significant directional shifts from baseline (*P<0.05; **P<0.01).

**Table 2:** Aftereffects of all of the single- and double-cursor trials in experiment 2.

Rotation (°)	−10	−5	0	5	10
−10	2.18** (0.44)	1.79** (0.41)	0.84* (0.37)	0.78 (0.42)	−0.56 (0.34)
−5	1.79** (0.41)	1.57** (0.38)	0.40 (0.40)	0.30 (0.35)	−0.70 (0.39)
0	0.84* (0.37)	0.40 (0.40)	0	−1.11 (0.36)	−1.38** (0.33)
5	0.78 (0.42)	0.30 (0.35)	−1.11 (0.36)	−2.96** (0.35)	−2.49** (0.37)
10	−0.56 (0.34)	−0.70 (0.39)	−1.38** (0.33)	−2.49** (0.37)	−3.49** (0.38)

The format is the same as that in Table 1.

Because of the nonlinear dependence of the aftereffects on the amount of the imposed visual rotation, *K_s_* decreased as the amount of visual rotation increased, indicating that the visual error information that was closer to the predicted movement was more effectively used for movement correction in the subsequent trial (Fig. 2B). There was a significant difference between *K_s_* values of each rotation (experiment 1, *F_5, 85_* = 2.81, *P*<0.05; experiment 2, *F_3, 36_* = 3.71, *P*<0.05). These results were consistent with the findings of a previous study that has demonstrated that the motor learning system estimates the relevance of each observed error and adapts strongly to more relevant errors [12].

There was an asymmetry in *K_s_* that depended on the direction of rotation. The values of *K_s_* for the identical amount of rotation were significantly greater when the rotations were in the positive (CCW) direction, as determined with two-way repeated-measures ANOVA (rotation angles × directions; *F*_1, 129_ = 7.59, *P*<0.01). The reason for this asymmetry was unclear, but we speculated that this was caused by the biomechanical characteristics of the arm. Due to the arm configuration, it is possible that reaching in the CCW direction was easier than reaching in the CW direction because, possibly, of the needed torque [13]. Thus, this might have made the movement correction in the CCW direction greater, although this idea needs to be tested further.

#### Aftereffects in the double-cursor condition

No significant difference was observed in the peak velocity of the subsequent trial of the visual perturbation trial (single-cursor trial, 471.8±1.89 mm/s; double-cursor trial, 472.5±1.37 mm/s; *t*_25_ = 0.27, *P* = 0.79). Therefore, the difference in the aftereffects between these conditions, if any, was not due to a difference in movement kinematics.

**Figure 3:**
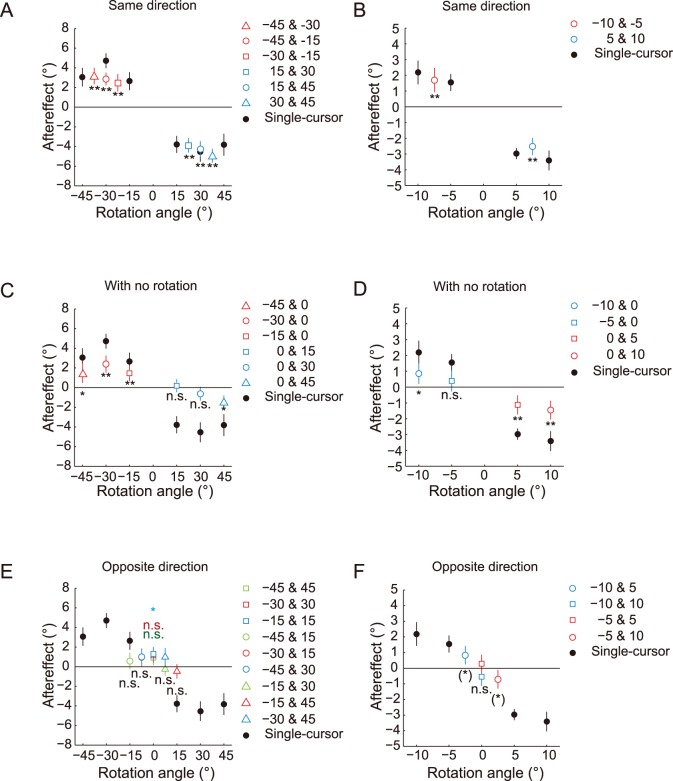
Averaged aftereffects of the double-cursor trials in experiments 1 (left) and 2 (right). The black circles in each panel indicate the aftereffects of the single-cursor trials for comparison. A, B: The aftereffects when two cursors were rotated in the same direction. The red and blue plots indicate the aftereffects of the double-cursor trials. An aftereffect is plotted at the center of two rotational angles (e.g., the aftereffect for the combination of −45° and 30° is plotted at 7.5° on the horizontal axis). C, D: The aftereffects when 1 cursor was not rotated. The open circles indicate the aftereffects of the double-cursor trials. The plot position corresponds to the rotated cursor (e.g., the aftereffect for the combination of 0° and 30° is plotted at 30° on the horizontal axis). E, F: The aftereffects when the cursors were rotated in the opposite directions. The green, red, and blue plots indicate the aftereffects of the double-cursors trials. An aftereffect is plotted at the center of 2 rotational angles. The asterisks indicate significant directional shifts from baseline (*P<0.05; **P<0.01). The statistical significance of the single-cursor trials is not shown. The error bars indicate ±1 SE.

When the visual perturbations were applied to the two cursors in the same direction (e.g., +15° and +30°), significant aftereffects were observed (Table 1 and Table 2; Fig. 3A, B). Even when one of the cursors was not rotated (i.e., 0°), we observed significant aftereffects in seven of the 10 combinations (Table 1 and Table 2; Fig. 3C, D), but the amplitude was smaller than in the single-cursor condition. Therefore, even when one of the two cursors followed the predicted movement exactly, the motor learning system was unable to ignore the presence of the other rotated cursor. In contrast, when the direction of the perturbation was in the opposite direction (e.g., +15° and −30°), the aftereffects were considerably reduced and not significant in almost all of the combinations (Table 1 and Table 2; Fig. 3E, F).

**Figure 4:**
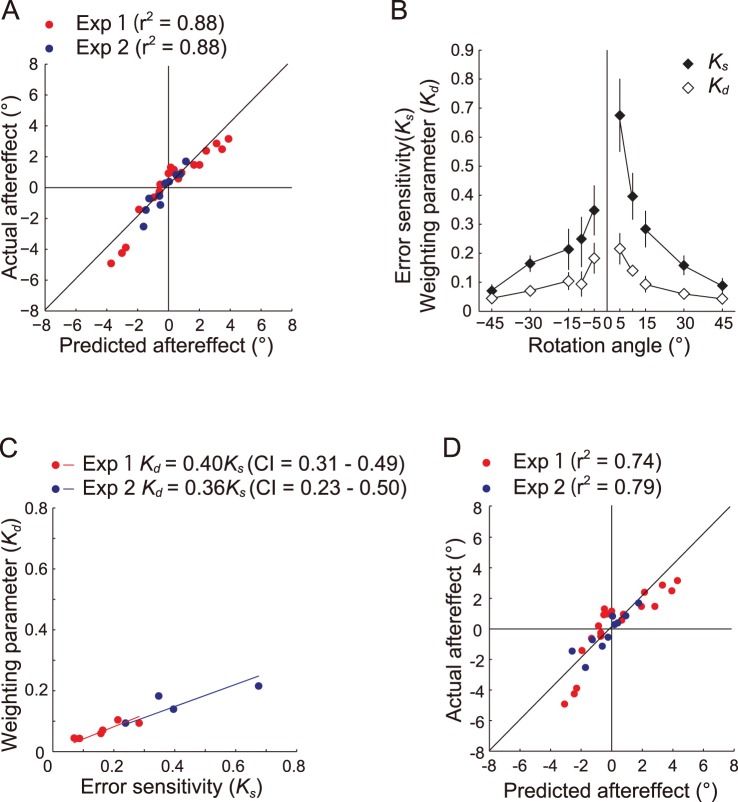
The results of experiments 1 and 2 are indicated in the same panel. A: The relationship between the aftereffects that were predicted by the linear integration model (eqs. 2 and 3) and the actual aftereffects. B: Comparisons between the error sensitivity (*K_s_*) of the single-cursor trials and the estimated weighting parameter (*K_d_*) of the double-cursor trials for each imposed visual rotation. The filled diamonds indicate *K_s_*, and the open diamonds indicate *K_d_*. Both *K_s_* and *K_d_* decayed as the magnitude of rotation increased, and *K_d_* was about 40% of the corresponding *K_s_*. The lines are disconnected between 10° and 15° because the data originated from different experiments. The error bars indicate ±1 SE. C: Linear regression between *K_d_* and the corresponding *K_s_*. The red plots indicate the parameters of experiment 1, and the blue plots indicate the parameters of experiment 2. The coefficients of regression and the confidence intervals (CI) are also shown. D: The relationship between the aftereffects that were predicted with the parameters that were estimated by leaving 1 cursor combination out at a time and the actual aftereffects.

In order to investigate how the information from the cursors was used to modify the movement direction in the subsequent trial, we estimated a weighting parameter (*K_d_*, eq. 2) for each imposed visual rotation. The linear integration model fit the actual data well (experiment 1, *r* ^2^ = 0.88; experiment 2, *r^2^* = 0.88; Fig. 4A). Two-way repeated-measures ANOVA (weighting parameter × rotation angle) showed that *K_d_* decreased with the increasing magnitude of rotation, as in the case of *K_s_* in the single-cursor trials (experiment 1, *F_5, 204_* = 4.38, *P*<0.01; experiment 2, *F_3, 96_ = *2.94, *P*<0.05; Fig. 4B). However, the main effect of the weighting parameter showed that the value of *K_d_* for each rotated cursor was significantly smaller (experiment 1, *F_1, 204_* = 3.89, *P*<0.01; experiment 2, *F_1, 96_ = *21.4, *P*<0.01) than the corresponding *K_s_*. The slopes of the linear regression between *K_d_* and *K_s_* were 0.40 (CI = 0.31 to 0.49) for experiment 1 and 0.36 (CI = 0.23 to 0.50) for experiment 2 (Fig. 4C).

#### Parameter estimation was not dependent on the cursor combinations

The model represented by eq. 2 assumed that the *K_d_* of one cursor remained constant, despite the visual rotation of another cursor. For example, the *K_d_* to the 15° rotated cursor was assumed to be constant regardless of the amount of rotation of another cursor (e.g., −15° or 45°). However, in contrast to this assumption, it is possible that the *K_d_* of one cursor was influenced by the visual rotation of another cursor (i.e., the *K_d_* to the 15° rotation was dependent on the amount of rotation of another cursor [i.e., −15° or 45°]). We examined the validity of the model of eq.2 using a leave-one-out cross validation procedure as follows. First, a weighting parameter (*K_d_*) was estimated for each participant by removing a certain cursor combination. As a result, we used 20 data points (experiment 1) and nine data points (experiment 2) of the averaged aftereffects from the double-cursor trials to estimate six (experiment 1) and four (experiment 2) parameters for the imposed visual rotations. Then, we predicted the aftereffect of the removed cursor combination with the estimated *K_d_*. This procedure was repeated for all 21 cursor combinations in experiment 1 and one of all 10 cursor combinations in experiment 2, so that 21 and 10 pairs of the predicted and the actual aftereffects were obtained, respectively. We found that there was a high correlation between the predicted and the actual aftereffects for experiment 1 (*r* ^2^ = 0.74; Fig. 4D) and for experiment 2 (*r* ^2^ = 0.79; Fig. 4D), indicating the validity of the weighted summation model (eq. 2).

#### Simultaneous utilization of two visual errors

A naïve idea of how the information from two cursors can be utilized is that, when two rotated cursors are displayed, the visuomotor learning system assumes that the rotation is imposed in the averaged direction. For example, when the cursors were rotated by −15° and 45°, the visuomotor learning system considered it as if a 15° [(−15° +45°)/2] rotation was imposed. However, the present data demonstrated that the aftereffects of these rotations were significantly smaller than the aftereffects of a 15° rotation (*t*_70_ = 4.91; *P*<0.01). Similar results were obtained with cursor combinations of 0° and 30° (*t*_71_ = 4.38; *P*<0.01) and 15° and −45° (*t*_70_ = 3.02; *P*<0.01). We failed to detect significant differences for the combinations of 0° and −30° (*t*_70_ = 0.50; *P* = 0.62) and 15° and 45° (*t_71_* = 0.43; *P* = 0.67), probably because the aftereffects in the double-cursor trials (the combination of 0° and −30°) were too small and because of the saturation of the aftereffects in the single-cursor trials (the combination of 15° and 45°).

#### Possibility of fifty-fifty utilization of one cursor

**Figure 5:**
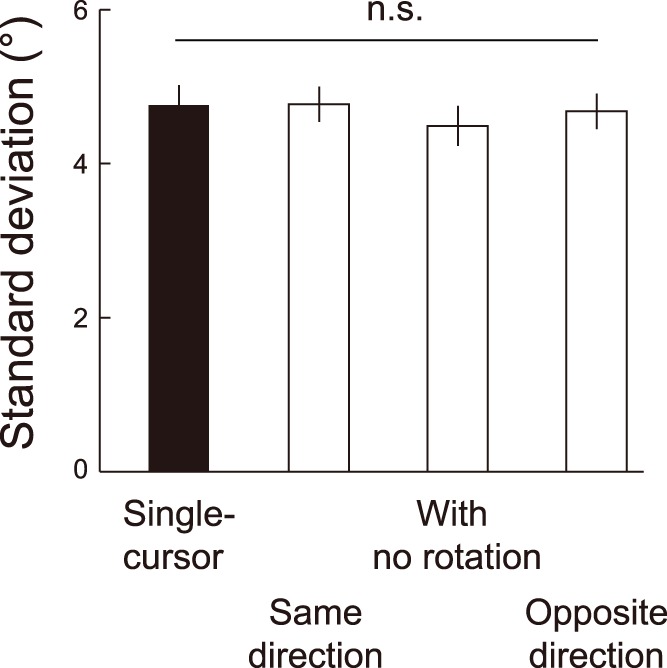
The relationship between the aftereffects and the mean value of two visual rotations in the double-cursor trials. The data are plotted for each of the differences in the angles between rotations. For example, the data for 45° (yellow) that is plotted at 22.5° is the aftereffect when the cursors were rotated by 0° and 45°. Note that the data for 0° is the aftereffect that was obtained in the single-cursor condition.

In contrast to the simultaneous utilization of two cursors, there was another possibility that the participants utilized only one of two cursors in a trial and the cursor that was utilized varied from trial to trial. For example, when the cursors were rotated by −30° and 30°, if the participants randomly utilized one of the two cursors in 50% of the trials, then the averaged value of the aftereffects would be almost 0°, as was obtained in our experiment. This was not compatible with the idea of the simultaneous utilization of two cursors. In order to examine this possibility, we compared the SDs of the aftereffects that were obtained from all participants among the single-cursor trials and the double-cursor trials in the same direction, without rotation, and in the opposite direction. If there was such a random utilization, the distribution of the aftereffects should have had two peaks and resulted in the increased SDs of the aftereffects. However, we found no significant differences in the SD of the aftereffects among these four conditions (*F_3, 37_* = 2.26, *P* = 0.85; Fig. 5), supporting the simultaneous utilization of two cursors in the double-cursor trials.

#### Averaged movement direction of cursors was not utilized

**Figure 6:**
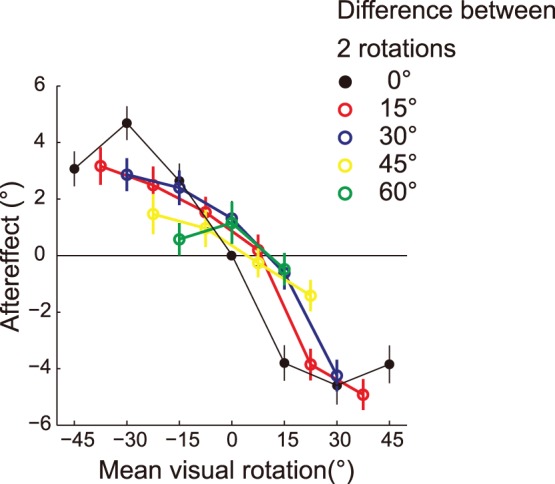
Lack of significant differences in standard deviations (SD) of the aftereffects that were obtained from all participants between the single- and the double-cursor trials. A black bar indicates the averaged SD of all of the single-cursor trials. The white bars indicate the averaged SD of the double-cursor trials of the same direction (“Same direction”), without rotation (“With no rotation”), and of the opposite direction (“Opposite direction”). The error bars indicate ±1 SE.

The spatial averaging of targets is one strategy that can be utilized when there are multiple movement goals for reaching [14]. However, in our paradigm, the results showed that it was unlikely that our motor system utilized the averaged direction of cursors as an error. Figure 6 indicates how the aftereffects were related to the mean value of the two visual rotations: each color plot corresponds to each of the differences in the angles between the two rotations (the data for 0° indicates the data for the single-cursor trial). As the difference in the angles becomes greater, the aftereffect to the mean value of the rotations becomes smaller and the deviation from the single-cursor trial becomes greater. Therefore, the motor learning system does not simply utilize the averaged visual error information to correct the subsequent movement direction.

### Experiment 3

#### Aftereffects and their relationship to rotations in the single-cursor condition

**Figure 7:**
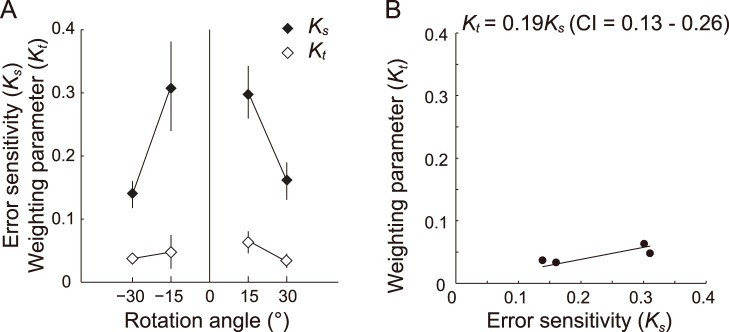
Comparisons between the error sensitivity (*K_s_*) in the single-cursor trials and the estimated weighting parameter (*K_t_*) in the triple-cursor trials for each imposed visual rotation. A: The filled diamonds indicate *K_s_*, and the open diamonds indicate *K_t_*. Both *K_s_* and *K_t_* decayed as the magnitude of rotation increased, and *K_t_* was further decreased from that in experiment 1. The error bars indicate ±1 SE. B: The linear regression between *K_t_* and the corresponding *K_s_*. The coefficients of regression and the confidence intervals (CI) are also shown.

Consistent with the results of experiments 1 and 2, significant aftereffects to all visual rotations were noted in the single-cursor trials (Table 3). As in experiments 1 and 2, we observed a decay pattern of *K_s_* with the increasing magnitude of the imposed visual rotations (Fig. 7A). The value of *K_s_* was similar between experiments 1 and 3. Indeed, there was no significant main effect between experiments in a two-way ANOVA (experiment × rotation angle; *F_1, 87_* = 0.77, *P = *0.39), confirming the reproducibility of *K_s_*.

**Table 3:** Aftereffects of all the single- and triple-cursor trials in experiment 3.

Rotation 1 (°)	−30					
Rotation 2 (°)	–	−15	−15	−15	0	
Rotation 3 (°)	–	0	15	30	15	
Aftereffect (°)	4.32** (0.42)	1.31** (0.40)	1.00* (0.38)	0.81 (0.42)	−0.05 (0.38)	
Rotation 1 (°)	−15			15		30
Rotation 2 (°)	–	0	15	–	0	–
Rotation 3 (°)	–	30	30	–	30	–
Aftereffect (°)	4.19** (0.44)	−0.40 (0.37)	−1.54** (0.35)	−4.03** (0.38)	−1.79** (0.33)	−4.57** (0.41)

The combinations of the three rotation angles of the cursors are shown in the first three rows (the blank in the second and third row indicates the single-cursor trial). The upper value of each cell of the aftereffect indicates the mean and the number in parentheses indicates 1 SE. The asterisks indicate significant directional shifts from baseline (*P<0.05; **P<0.01).

#### Aftereffects in the triple-cursor condition

**Figure 8:**
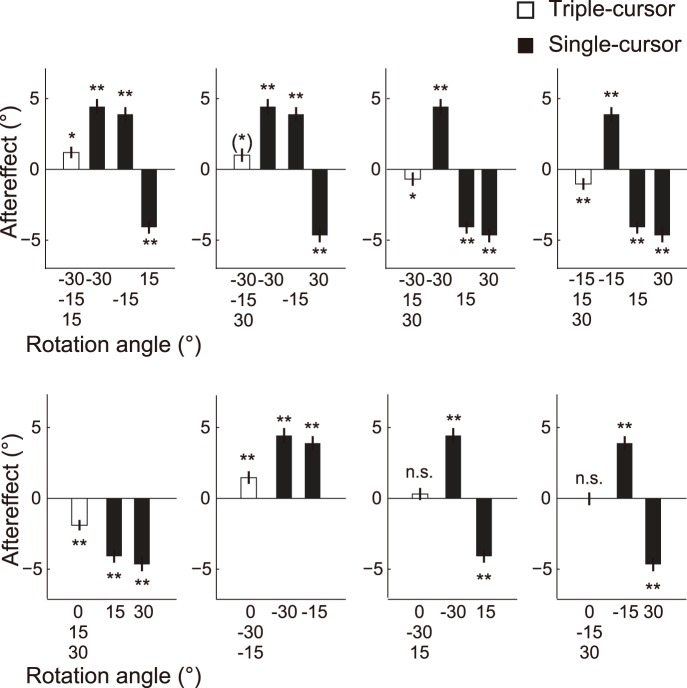
The aftereffects of experiment 3. The black bars indicate the aftereffects of the single-cursor trials of the labeled rotations, and the white bars indicate the aftereffects of the triple-cursor trials when the labeled rotations were simultaneously imposed. The data for 0° are not shown in the lower row because the average of the standardized baseline movement directions is always 0°. The asterisks indicate significant directional shifts from baseline [*P<0.05; **P<0.01; (*)*P*<0.05 in one-tailed tests]. The error bars indicate ±1 SE.

Significant aftereffects were observed in the triple-cursor trials in all cases, except for the combination in which the three cursors were rotated by −30°, −15°, and 30° (Table 3; Fig. 8). The aftereffects were also significant when one of them was not rotated and the directions of the two rotations were the same (Table 3; Fig. 8). However, the aftereffects were not significant if one of them was not rotated and the directions of the two rotations were the opposite (Table 3; Fig. 8).

The linear integration model (eq. 3) fit the data well (*r* ^2^ = 0.98), as in the cases of experiments 1 and 2. The estimated weighting parameter (*K_t_*) decreased with the increasing magnitude of the imposed visual rotations (Fig. 7A), as was observed for *K_s_* and *K_d_* in experiments 1 and 2 (Fig. 4B). Two-way repeated-measures ANOVA (weighting parameter × rotation angle) detected significant main effects for the weighting parameter (*F_1, 96_* = 56.7, *P*<0.01), the rotation angle (*F_3, 96_* = 4.47, *P*<0.01), and the interaction (*F_3, 96_* = 2.73, *P*<0.01). A post hoc test indicated that there was a significant difference in *K_s_* for different absolute amounts (e.g., combinations of −10° and −5° or −10° and 5°), whereas *K_t_* was not significantly different among the angles.

The coefficient of linear regression between *K_t_* and *K_s_* was 0.19 (*P*<0.01), and the CI was 0.13 to 0.26 (Fig. 7B). Therefore, *K_t_* decreased considerably from the corresponding coefficient (*K_d_*) in the double-cursor trials.

## Discussion

When we learn to perform a desired movement in various environments, the central nervous system gradually develops an internal representation, or an internal model, of the movements in order to predict a movement consequence from a motor command [1]–[4]. The internal model is constructed on the basis of error information between the sensory prediction from the motor command and the actual sensory consequence, indicating the importance of their proper association. However, such proper association is not always guaranteed. For example, the brain faces the difficulty of assigning responsibility for an error to a specific limb when a cursor is manipulated with both hands [8], [9]. The results of our previous study have shown that, when visual information from only one hand was provided by a cursor during bimanual visuomotor learning, the visual rotation that was imposed on the cursor lead to the correction of not only the relevant hand’s movement, but also the opposite hand’s movement, even though the participants knew that only the relevant hand was involved in moving the cursor [8]. The explicit knowledge about the relationship between the relevant hand and the cursor did not prevent the other hand from adapting to the error, suggesting the implicit nature [10] of the credit assignment process, in which two hand controllers utilize one error information simultaneously for motor learning.

In the present study, we aimed to explore this automatic property of the credit assignment process in depth. Specifically, when we observed multiple movement errors that resulted from a single action, we wanted to determine how the motor learning system assigned the error information to correct the movement in the subsequent trial. We asked, “Does this system just ignore one of the errors, or does it ignore both and consider the averaged direction of the cursors as an error? Or, alternatively, does it use the information from both errors simultaneously?” In order to examine this problem, we quantified the visuomotor adaptation when two or three visual errors of different angels were generated simultaneously. We found that multiple errors were likely to be simultaneously assigned to a single movement controller and that the aftereffects in the subsequent trial were normalized between the aftereffects to the respective errors. We used the word “normalize” instead of “average” because such an effect was similar to the divisive normalization mechanism that has been shown in the neural computation that occurs during visual information processing [15]. The normalization indicated that all errors were partially utilized at the same time in an integrated manner because the relationship between the aftereffects and the errors could be described well by a simple linear integration model consisting of a weighted sum (not just a summation) of the aftereffects to the respective errors.

When we observed multiple cursors that were simultaneously moving with an action, we were perplexed as to how we should perform the subsequent movement. However, even in such a situation, the visuomotor learning system can provide a solution that is based on the implicit processing of the visual error information.

### Dependence of the Aftereffects on the Magnitude of Error

In the single-cursor trials in experiments 1–3, we found that the aftereffects were not proportional to the magnitude of rotation (i.e., the size of the error) but appeared to be saturated as the degree of rotation became greater (Fig. 2A). Accordingly, the error sensitivity decayed with the increasing magnitude of the imposed visual rotations (Fig. 2B; Fig. 7A), which was consistent with the results of previous studies that have examined the adaptation to external force [16], [17] and visual disturbance [12], [18]. Therefore, the smaller the size of the error (i.e., the difference between the visual feedback of the movement consequence and what is predicted by the internal model), the more the movement error is utilized to correct the subsequent movement.

Wei and Körding [12] have suggested that the decrement in sensitivity that occurs with the amount of visual disturbance is explainable by a Bayesian integration process between visual and proprioceptive information. As the visual rotation becomes greater, the motor system relies more on proprioception, resulting in the decreased sensitivity of the movement correction to the visual rotation. Another computational study [19] has suggested that the movement error modifies the primitives for motor learning that correspond to the movement error direction and not to the planned movement direction. Accordingly, when the visual error is greater, the difference in the population of the primitives between the planned movement direction and the movement error direction becomes greater. This might lead to a decrement in adaptation because the degree of modification of the primitives for the planned movement direction might decrease.

### Aftereffects when 2 or 3 Cursors are Displayed

In the double-cursor trials (experiments 1 and 2), when the directions of the perturbations were the same (e.g., 15° and 45°), significant aftereffects were observed in the opposite direction (Fig. 3A, B). In contrast, when the directions of the perturbations were the opposite (e.g., 30° and −15°), the aftereffects were not significant in almost all of the combinations. Interestingly, when one cursor was not rotated (e.g., 0° and 30°), significant aftereffects were still observed in some combinations (Fig. 3C, D). Therefore, the visuomotor learning system did not ignore the rotated cursor even when the other cursor followed exactly the predicted movement direction (i.e., 0°).

These results implied that the visuomotor learning system utilized the information from the two cursors simultaneously. In order to evaluate the manner of integration, we assumed that the aftereffects were determined by a linear integration model of the imposed rotations (eqs. 2, 3) and that each amount of rotation had a specific coefficient (*K_d_* and *K_t_*). *K_d_* was 37% (obtained in the double-cursor trials) and *K_t_* was 19% (obtained in the triple-cursor trials) of the corresponding *K_s_* (obtained in the single-cursor trials; Fig. 4C, 7B). The similarities in the decay patterns among *K_s_*, *K_d_*, and *K_t_* implied that the manner of usage of the visual rotation information was preserved, and the reduction in the value with the number of cursors indicated that the response was not simply summed but that some normalization process might be at work.

### Validity of the Linear Integration Model

Although our analysis showed that the parameters that were estimated by the linear integration model described the empirical results well, it was possible that a weighting parameter of a particular cursor was drastically changed according to the rotation of the other cursor. In order to examine this notion, we conducted a kind of cross-validation for the linear model by estimating weighting parameters of the cursors, while leaving one cursor combination out at a time. The results showed that, even if we excluded a particular cursor combination, we could correctly predict the aftereffects of the corresponding cursor combination (Fig. 4D). In addition, the data suggested that, during the double-cursor trial, the error sensitivity of a cursor was not influenced by the rotation of the other cursor.

### Were Multiple Cursors Utilized Simultaneously?

A critical question was whether multiple errors were really utilized simultaneously to correct the subsequent movement. Indeed, the results of the double-cursor trials can also be explained by assuming that the participants utilized only one of the two cursors in a trial and the cursor that was utilized varied from trial to trial. If there was such a utilization, the aftereffects should have had two peaks around the aftereffects to the −30° and 30° rotations, rather than at 0°, and, as a result, the variability of the aftereffects should have increased. However, we did not find any evidence that the SD was greater in the double-cursor trials (Fig. 5), excluding the possibility that one of the two cursors was randomly utilized in a trial.

Wei and Körding [20] have also examined how perturbations to the visual feedback that is displayed by a dot just after a trial induces aftereffects in the subsequent trial. They not only biased the hand’s position, but also manipulated the uncertainty of the visual feedback by presenting five dots, and they demonstrated that the larger the uncertainty of the visual feedback (i.e., the variance of the dots), the smaller is the adaptation rate. Interestingly, their observations were similar to ours if we regard the difference in the angles between the two cursor-s as the index of uncertainty (Fig. 5). They have explained their results by assuming that the motor learning system uses the uncertainty of the feedback in estimating the state of the limb in an optimal way. An intriguing challenge would be to consider how our mechanistic interpretation of the usage of error information was related to their stochastic interpretation.

### Possible Neuronal Mechanisms

The present results implied that the visuomotor learning system processed multiple visual errors simultaneously and integrated them not by simple summation but by normalization. As for the simultaneous processing of visual information, the neuronal process of decision making in the presence of several action choices (e.g., several targets) has been rigorously investigated [21]–[27]. For example, when two visual targets are provided together at the initial phase of motor preparation, both potential targets may be simultaneously represented in the dorsal stream of the visual process before the target to be reached is selected [21]. In addition, such parallel neuronal processing of visual information might affect behavior; for example, when a distractor is added to a target for saccadic movement with a short latency, the eye trajectory deviates toward the distractor [28]. Tipper et al. [29] have suggested that the weighted average of the activities of the neural populations that encode either the direction of the target or the distractor represents the terminal direction of the movement according to the population coding theory. Therefore, the resultant direction will be located halfway between the target and the distractor [30]. This indicates that multiple movement goals are represented in parallel and averaged when a motor command is outputted.

In addition to the parallel processing of multiple pieces of visual information, normalization might also occur because the aftereffects to the double-cursor trials were not summed but were close to the averaged value of each aftereffect that was observed when one cursor was separately presented. Such normalized responses to sensory information have also been found in several other biological systems, including the visual system. For example, studies have shown that the population response of V1 neurons to multiple visual stimuli is not the simple summation of the responses to each stimulus but is effectively normalized depending on the number of neurons [15], [31], [32]. Recent studies have indicated that such normalized neuronal responses to multiple visual objects are caused by dispersed attention [33].

During the adaptation to multiple cursors in the present study, such normalized responses of neurons in visual areas might have been involved in the normalized aftereffects. Considering the contribution of the cerebellum [4], [34]–[37] and the posterior parietal cortex [26], [38], [39] to visuomotor learning, the neurons in these areas are also likely to exhibit normalized responses when multiple cursors are displayed. Additional studies are required to clarify which areas are involved in the integration of multiple pieces of visual error information.

### Effects of Visual Attention

One question remaining from our study may be whether decreased adaptation can be accounted for by decreased visual attention to errors that are caused by the display of multiple errors. The visual attention and visual error utilization that we aimed to investigate in the present study may not be the same because it is nontrivial that errors that are more attended to are utilized more to correct movement by the motor learning process. We cannot assess how the level of visual attention to errors influenced the adaptation with the current experiments. One reason for this is that we did not measure eye movements during the experiments. However, we expect that there is an influence of attention on the credit assignment process during motor learning because, for example, some parts of the posterior parietal cortex are involved both in movement error processing during reach adaptation [38] and in the dorsal attention network [40], [41]. Further investigations are needed to clarify the relationship between visual attention and visual error processing during motor learning by both behavioral and neurophysiological approaches.
